# Evolution of competitive ability and the response to nutrient availability: a resurrection study with the calcareous grassland herb, *Leontodon hispidus*

**DOI:** 10.1007/s00442-024-05657-1

**Published:** 2025-01-04

**Authors:** Pascal Karitter, Emma Corvers, Marie Karrenbauer, Martí March-Salas, Bojana Stojanova, Andreas Ensslin, Robert Rauschkolb, Sandrine Godefroid, J. F. Scheepens

**Affiliations:** 1 Plant Evolutionary Ecology, Institute of Ecology, Evolution and Diversity, Faculty of Biological Sciences, Goethe University Frankfurt, Max-Von-Laue-Str. 13, 60438 Frankfurt am Main, Germany; 2 Area of Biodiversity and Conservation, Department of Biology and Geology, Physics and Inorganic Chemistry, University Rey Juan Carlos-ESCET, Tulipán S/N., 28933 Móstoles, Madrid Spain; 3 Department of Biology and Ecology, Faculty of Science, University of Ostrava, Chittussiho 10, 710 00 Slezská Ostrava, Czech Republic; 4 Conservatory and Botanic Garden of the City of Geneva, Chemin de L´Impératrice 16 1, 1296 Chambésy, Geneva Switzerland; 5 Department of Plant Biodiversity, Institute of Ecology and Evolution With Herbarium Haussknecht and Botanical Garden, Friedrich Schiller University Jena, Jena, Germany; 6 German Centre for Integrative Biodiversity Research (iDiv) Halle-Jena-Leipzig, Leipzig, Germany; 7 Meise Botanic Garden, Nieuwelaan 38, 1860 Meise, Belgium; 8 Instituto de Investigación en Cambio Global (IICG-URJC), Universidad Rey Juan Carlos, Móstoles, Madrid, Spain

**Keywords:** Competition, Fertilization experiment, Global change, Rapid evolution, Resurrection approach

## Abstract

Rapid environmental changes across Europe include warmer and increasingly variable temperatures, changes in soil nutrient availability, and pollinator decline. These abiotic and biotic changes can affect natural plant populations and force them to optimize resource use against competitors. To date, the evolution of competitive ability in the context of changes in nutrient availability remains understudied. In this study, we investigated whether the common calcareous grassland herb *Leontodon hispidus* recently evolved its competitive ability and response to nutrient availability. We compared ancestors sampled in 1995 and descendants sampled in 2018 and applied a competition treatment in combination with weekly nutrient treatments (no fertilizer, nitrogen, phosphorus, and both). We found evidence for evolution of increased competitive ability, with descendants producing more vegetative biomass than ancestors when grown under competition. Furthermore, supplementing nutrients (especially N) reduced differences in competitive ability between ancestors and descendants, suggesting that nutrients are a limiting factor in interspecific competition, which could be linked to the decreasing nitrogen emissions into the atmosphere since the 1990s. Our study demonstrates rapid contemporary evolution of competitive ability, but also the complexity of the underlying processes of contemporary evolution, and sheds light on the importance of understudied potential selection agents such as nutrient availability.

**Supplementary Information:**

The online version contains supplementary material available at 10.1007/s00442-024-05657-1.

## Introduction

Environmental conditions have been rapidly changing for decades and are affecting ecosystems worldwide (IPCC 2018). These rapid changes include, among others, higher frequencies and intensities of droughts and heatwaves (Dore 2005; Ruosteenoja et al. 2018; Samaniego et al. 2018), pollinator decline (Potts et al. 2010), and changes in nutrient availability (Newman 1995; Smith et al. 1999; Galloway et al. 2008). These abiotic and biotic changes can disturb natural plant populations by imposing significant selection pressures and forcing plants toward optimization of resource use in presence of competitors (Mosquin 1971; Bonser and Ladd 2011; Gao et al. 2022).

Agricultural land use and fossil fuel combustion contribute to the continuous release of nitrogen (N) and phosphorus (P) into ecosystems worldwide through extensive fertilization and deposition from the atmosphere (Newman 1995; Smith et al. 1999; Galloway et al. 2008). Excess agricultural fertilizer can be released to adjacent ecosystems via runoff or transport by freshwater bodies (Ceulemans et al. 2014). Since the beginning of the industrial revolution, the yearly release of N in the biosphere increased from 15.3 to 259 Mt and of P from 0.3 to 16 Mt (Peñuelas et al. 2012). Whereas N emissions have been steadily decreasing since the 1990s (European Environment Agency 2021), P levels are still above the recommended ranges in many agricultural soils in Europe (Djodjic et al. 2004; BDB 2005; Ketterings et al. 2005; Reijneveld et al. 2010). Phosphorus has a much slower amelioration over time than N, and thus, the effects of P enrichment are also likely to be more persistent in future (Parkhurst et al. 2022). These shifts in the availability of N and P are likely affecting plant populations, and rapid adaptation to those changes will be essential for population persistence (Sala et al. 2000; Tilman et al. 2001). While the impact of Nitrogen excess on plants has been widely studied since decades, especially through atmospheric N deposition (Bobbink et al. 1998; Stevens et al. 2004; Phoenix et al. 2006; Clark and Tilman 2008; Conley et al. 2009; Cleland and Harpole 2010), the effect of P enrichment has received less attention (but see Janssens et al. 1998; Ceulemans et al. 2011, 2014; van Dobben et al. 2017). Nutrient enrichment can have a strong impact on plant populations causing competitive exclusion, higher susceptibility to pests and abiotic stressors, soil acidification, and even toxicity (Hautier et al. 2009; Johnson 1993; Olsson and Tyler 2004; Bobbink et al. 2010; Stevens et al. 2010a, b).

Since plants are continuously competing for space and resources, such as light, water, and nutrients (Craine and Dybzinski 2013), changes in the availability of these resources may affect the evolution of plant responses as less competitive species are likely to experience higher mortality (Grime 1973). An increase in soil nutrient resources in a nutrient-limited habitat will generally stimulate increased aboveground vegetative growth, but will also reduce light availability for smaller plants as competitors outgrow them. In response to shading, plants can respond plastically to reach more light (shade-avoidance syndrome, SAS) and to stay competitive. Among those responses are the acceleration of flowering time, production of more erect leaves in rosette plants and their elongation (Casal 2012). In naturally nutrient-poor habitats, competition may shift from below- to above-ground when nutrient concentration suddenly increases (Hautier et al. 2009), whereas a reduction in nutrient concentration will result in stronger belowground competition (Newman 1973). In the latter scenario, plants may increase their root length to acquire more nutrients for themselves while reducing the nutrient availability for their competitors (i.e., supply pre-emption, Craine et al. 2005). Nutrient availability is also affected by drought episodes which are becoming more frequent with ongoing climate changes. This is because the root uptake of most mineral nutrients depends on soil moisture and hence, a reduction of soil moisture may limit nutrient uptake (Taiz and Zeiger 2006). Additionally, the enzymatic activity of soil microorganisms may also be affected by droughts, leading to impairment of nutrient mineralization (Silva et al. 2010). Therefore, unfavorable soil physicochemical soil properties can impede nutrients uptake despite their high soil concentration (Amtmann and Blatt 2009). Consequently, changes in nutrient supply and resulting impacts on competition can impose strong selection pressure on plants to increase either their stress tolerance or competitive ability by adjusting growth-related traits under the novel environmental conditions (Falster and Westoby 2003; Craine and Dybzinski 2013). Given the strong degradation of natural habitats by nutrient enrichment and the resulting increase in competition, understanding the ability of plant populations to adapt to changes in nutrient availability is of high importance (Hautier et al. 2009; Smith et al. 1999; Stevens et al. 2010a, b; Ceulemans et al. 2013, 2014).

Over the past three decades, the resurrection approach has been widely used to study rapid evolution of plant populations (Thomann et al. 2015a, b; Franks et al. 2018; Wooliver et al. 2020; Hamann et al. 2021; Rauschkolb et al. 2023; Karitter et al. 2024). This approach involves an experimental design that utilizes seeds collected from a population before (ancestors) and after (descendants) a potential selection pressure acted on the population, such as consecutive drought years. Comparisons of the phenotypes of these two generations in a controlled environment can then uncover evolutionary changes (Franks et al. 2007). Resurrection studies have provided compelling evidence that plant populations can undergo rapid evolution in various morphological, physiological, and phenological traits within a few generations (Franks et al. 2007; Nevo et al. 2012; Thompson et al. 2013; Thomann et al. 2015a, b; Hamann et al. 2018; Sekor and Franks 2018). Whereas the evolution of competitive ability has been studied in some resurrection experiments (Sultan et al. 2013; Frachon et al. 2017; Ziska 2017), resurrection studies on evolutionary responses to nutrient availability are currently, to our knowledge, lacking. Sultan et al. (2013) conducted a resurrection study on the invasive species *Polygonum cespitosum* and found evolution of higher competitive ability after 11 years through higher reproductive output, and stronger plasticity in physiological traits and root allocation. Frachon et al. (2017) found that *Arabidopsis thaliana* responded to local warming and increased competition through a delay in bolting time and evolution of an adaptive strategy that mainly involved the tendency to escape competition in crowded environments through lateral growth. Since competitive ability is also highly dependent on abiotic factors, it is important to examine the evolution of competitive ability in the context of changing nutrient availability to gain a deeper understanding of plant responses to environmental changes.

We conducted a resurrection study to investigate recent adaptive evolution of *Leontodon hispidus* (Asteraceae)*,* a common herb in calcareous grasslands, to N and P enrichment and to competition. In calcareous grasslands, biodiversity is threatened by the increasing dominance of the grass *Brachypodium pinnatum* (Bobbink and Willems 1987; Ba̧ba 2003; Canals et al. 2017) and the evolution of increased competitive ability could be essential for the persistence of some plant populations in this habitat. We used ancestors sampled in 1995 and descendants sampled in 2018 (i.e., 23 years apart) of one population in a Belgian nature reserve. After two refresher generations, we grew ancestors and descendants under common conditions and applied a competition treatment using the natural competitor *Brachypodium pinnatum* (Poaceae). Furthermore, we applied nutrient treatments to plants subjected to competition, supplying those plants weekly with either no nutrients, or with nitrogen, phosphorous, or both. We measured growth, leaf, and floral traits. We hypothesized that the decrease in soil nutrient availability led to a shift from aboveground competition for light to belowground competition for nutrients. Thus, we expect evolution of lower competitive ability aboveground and higher competitive ability belowground. Further, we hypothesized that the decrease of N emissions over the last decades have selected for higher fitness in descendants of *L. hispidus* under low N availability. In contrast, we expect that descendants and ancestors respond similarly to high P availability due to slower reduction of P emissions and greater P soil persistence over the past two decades.

## Materials and methods

### Study species and seed origin

*Leontodon hispidus* L. (Asteraceae) is a perennial, rosette-forming, herbaceous plant. It is self-incompatible and can flower in the first year after germination, which typically occurs from June to October (Kühn and Klotz 2002). It is widespread throughout Europe and commonly found in calcareous grasslands, which received conservation priority by the European commission ("Festuco-Brometalia"; EU code 6210: Semi-natural dry grasslands and scrubland facies on calcareous substrates). Calcareous grasslands are threatened by eutrophication and lack of management (Habel et al. 2013) and as a typical species for this habitat *L. hispidus* is steadily declining in the northern parts of Belgium (Hoste et al. 2006).

Seed material was collected in one population in a nature reserve called “Thier à la Tombe” in the northeastern part of Belgium (50° 47′ 34.7" N, 5° 40′ 22.6" E) in 1995 (ancestors) and 2018 (descendants). The vegetation is a calcareous grassland that was unmanaged until 2007, after which sheep grazing was introduced yearly in spring and early summer. The nature reserve is situated on a west-facing slope next to an agricultural field. The distance to the nearest other population is approximately 2 km, decreasing the likelihood of cross-pollination between populations of *L. hispidus*. The ancestral seeds were collected by the Meise Botanic Garden (Belgium) for conservation purposes. Although the precise number of sampled individuals was not recorded, efforts were made to represent the genetic diversity of the population in the sampling. All seeds were cleaned, bulked, and dried at 15% relative humidity. The seeds were stored at − 20 °C in the seed bank of Meise Botanic Garden. In summer 2018, we revisited the population and collected the seeds from all inflorescences from 20 mother plants. The seeds were cleaned, bulked, and then stored at 4 °C. Analyses of the genetic diversity showed comparable levels of allelic richness and within-temporal-origin relatedness, indicating sufficient sampling efforts in both temporal origins and the absence of random evolutionary processes. (Rauschkolb et al. 2022).

### Experimental design

Both ancestral and descendant seeds were grown for a refresher generation (Rauschkolb, et al. 2022) in order to reduce environmental, maternal, and storage effects (Franks et al. 2018).

We sowed 300 seeds from each temporal origin and selected 15 random individuals per temporal origin that were haphazardly pollinated by hand in pollinator isolation cages to prevent unintentional cross-pollination (Rauschkolb, et al. 2022). The germination success of ancestral seeds was 93%, and thus, Indicating low likelihood for the occurrence of selective mortality during storage (invisible fraction, sensu Weis 2018). To increase the number of seeds per family, the F1 seeds were grown in the same conditions and the plants were pollinated within temporal accessions using commercial bumblebees. We cultivated the plants in the same conditions, and we used bumblebees (Natupol seeds, Koppert GmbH, Straelen, Germany) as pollinators. Ultimately, nine seed families from both ancestral and descendant temporal origin yielded sufficient seed material for the experiment.

In March 2022, we prepared 25 pots (1.5 L) for each maternal line with nutrient-poor soil (Einheitserde Typ 1, Einheitserde, Sinntal-Altengronau, Germany) in the greenhouse and sowed 3 seeds into each pot. Simultaneously, we sowed 150 g of seeds of *Brachypodium pinnatum* (UG12, Rieger Hofmann GmbH, Blaufelden-Raboldshausen, Germany) in 6 trays using the same nutrient-poor soil. *Brachypodium pinnatum* was used as a competing grass in this experiment as it is a natural competitor of *L. hispidus* in its natural habitat. All pots and trays were watered three times a week to soil capacity. Once the *L. hispidus* seedlings emerged and all seedlings developed their first true leaf, we thinned them to a single individual per pot and moved this individual to the center of the pot. Three weeks after germination, we started the nutrient and competition treatments. To prevent nutrient deficiencies, all pots were supplemented with 1.2 g of slow-release fertilizer (Osmocote Pro, Controlled Release Fertilizer 3–4, ICL Group, Ludwigshafen, Germany).

**Fig. 1:**
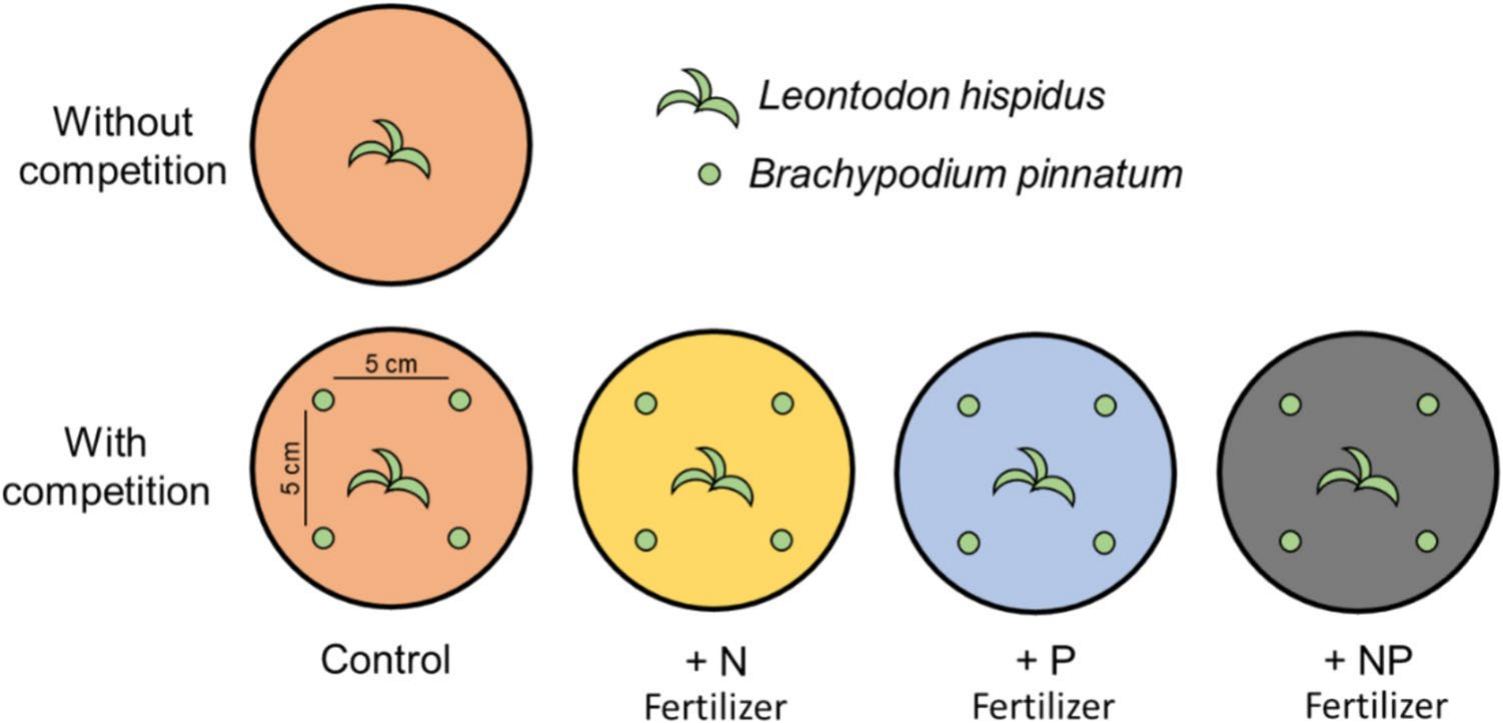
Experimental design of the study. Ancestors and descendants of *Leontodon hispidus* were cultivated in pots and divided into 5 treatment groups. One group was cultivated without competition and no additional nutrient supply. The other four groups were all grown with competition in combination with a weekly nutrient treatment (control, N fertilizer, P fertilizer, NP fertilizer). For the competition treatments, we used *Brachypodium pinnatum* which naturally occurs in the habitat of *L. hispidus* and is one of its strongest competitors. Each competition treatment involved the transplantation of four individuals of c. 10 cm tall *B. pinnatum* plants around *L. hispidus* in the center with an equidistance of 5 cm once the *L. hispidus* plants developed their first true leaves

We divided the pots into 5 treatment groups with 5 replicates per seed family and applied the following competition and nutrient treatments: (i) without competition and without fertilizer (i.e., without competition control); (ii) with competition and without fertilizer (with competition control); (iii) with competition and nitrogen fertilizer (N); (iv) with competition and phosphorus fertilizer (P); (v) with competition and nitrogen + phosphorus fertilizer (NP) (Fig. 1). In the competition groups, we transplanted four individuals of *B. pinnatum* with approximately 10 cm height into each pot with an equidistance of 5 cm around the center of the pot (Fig. 1). For the N source, we used urea (CH_4_N_2_O, Roth, Karlsruhe, Germany) and for the P source, we used monosodium phosphate (NaH_2_PO_4_, Roth, Karlsruhe, Germany). We chose these fertilizers, as they only contain the macronutrient of interest and no additional macronutrients (Marschner 1995). The plants were watered three times per week to soil capacity and weekly with their respective fertilizer solution to simulate constant nutrient influx: 17.86 mg urea (≈ 10 mg N) in 20 ml H_2_O for the N treatment; 21.92 mg monosodium phosphate (≈ 5 mg P) in 20 ml H_2_O for the P treatment. These concentrations were chosen as they simulate a strong influx of nutrients, which is comparable to the yearly influx of nutrients into ecosystems: 17 kg N/ha/year and up to 5 kg/P/ha/year (Newman 1995; Stevens et al. 2004). Plants in the NP treatment received the N and P treatment consecutively. In total, the experiment consisted of 450 pots (2 temporal origins × 9 seed families × 5 treatment groups × 5 replicates). We randomized all pots every two weeks and moved the pots to an outdoor common garden after 4 weeks.

### Plant measurements

During the experiment, we recorded the onset of flowering and the height of the first flower stem of *L. hispidus* every Monday, Wednesday, and Friday. We defined onset of flowering as the day when the first anther became visible. After 17 weeks, all plants had flowered, and we harvested them after measuring the rosette diameter. We counted and collected all the flower heads (Online Resource 1) and stems as reproductive biomass and the leaves as vegetative biomass. For each individual, three randomly selected healthy and fully developed leaves were sampled, and their combined area was measured with the smartphone application “easy leaf area free” (Easlon and Bloom 2014). The leaves were dried in a drying oven at 60 °C for three days and then weighed at a high-precision scale (CPA225D-0CE, *e* = 1 mg, Sartorius AG, Göttingen, Germany). We calculated specific leaf area (SLA) by dividing the combined leaf area by its dry weight. The root biomass of *L. hispidus* was separated from the roots of the grasses and washed to remove the soil. The root biomass, vegetative biomass, and reproductive biomass were separately dried in a drying oven at 60 °C for 72 h and then weighed at the high-precision scale as well. For the final values of vegetative biomass, we added the dry weight of the three leaves we collected for the leaf area measurements. Finally, we calculated reproductive investment as the ratio of reproductive biomass to vegetative biomass.

### Soil analysis

In the autumn of 2021, we took soil samples of 25 cm^3^ at 10 cm soil depth at four random locations in the natural population of *L. hispidus*. All four soil samples were bulked and dried at 40 °C for one week in a drying oven. We sieved the samples to < 2 mm, and we milled 0.3–1 g of the sieved soil with a Mixer Mill MM400 (Retsch, Haan, Germany) for 60 s with 30 rounds per second. To avoid contamination between samples, we cleaned the sieving and milling tools between samples with an air-compressor and water. The samples were then analyzed to determine the amount of fundamental minerals (total element content of P, K, S, Ca, C, N, and S), as well as pH level and salinity (Online Resource 2). Total C and N measurements were performed by elemental analysis through thermal combustion and thermal conductivity detection of CO_2_/N_2_ (Thermo Scientific, Flash 2000 HT Plus, Bremen, Germany). For total element concentrations, we digested the samples with a mixture of HNO_3_, HF, and H_2_O_2_ (4:2:1) in a microwave oven (Mars 6, CEM, Kamp-Lintfort, Germany). Then we complexed excess HF with H_3_BO_3_ and measured total element concentrations by ICP-OES. We confirmed complete element recovery of total digestions with certified reference material (BCR2, Columbia River basalt).

### Data analysis

Since we were specifically interested in the effects of competition per se and of the nutrient treatments per se, we divided and analyzed the data in two subsets. To analyze the effect of competition on the temporal origins, we included only the groups without fertilizer (i.e., without competition control, and with competition control) in the first subset. The second subset contained all groups with nutrient treatments (N, P, and NP) and the competition group without fertilizer (with competition control). All statistical analyses were performed using R (version 4.0.3, R Core Team 2020). We performed linear mixed effects models (LMMs) using the *lmer* function implemented in the *lme4* package (Bates et al. 2015) to analyze the following response variables: vegetative biomass, rosette diameter, root biomass, SLA, reproductive biomass, reproductive investment, flower stem height, and onset of flowering. Using the competition data set, we tested for effects of the competition treatment, temporal origin, and their interaction as fixed factors and seed family nested in temporal origin as random factor. Using the nutrient treatment data set, we tested for effects of the nutrient treatment, temporal origin, and their interaction as fixed factors and seed family nested in temporal origin as random factor. When the normality and homoscedasticity of model residuals were not met, we applied appropriate transformations to the response variables (see transformations in Tables 1 and 2). All LMMs were analyzed using the *ANOVA* function (Type I), and analyses were always followed by Tukey post-hoc tests for each treatment pair within temporal origins and for each temporal origin within each treatment using the *emmeans* package (Lenth 2021). We calculated marginal *R*^2^ (*R*^2^_m_) and conditional *R*^2^ (*R*^2^_c_) for these LMMs using the *r2* function of the *performance* package (Lüdecke et al. 2021) in order to assess the variance explained by the random factors (Online Resource 3).

**Table 1:** Results of the statistical models testing the effects of temporal origin (ancestors, descendants), competition (with, without) and their interaction on the response variables (y) vegetative biomass, rosette diameter, root biomass, specific leaf area (SLA), reproductive biomass, reproductive investment, flower stem height and onset of flowering of *Leontodon hispidus*

Response variable	Transformation	Explanatory variable	*df*	*F* value	*P* value
Vegetative biomass	sqrt(y)	Origin	1	7.12	**0.050**
		Competition	1	281.70	** < 0.001**
		Origin × competition	1	3.93	**0.038**
Rosette diameter	(y)^3^	Origin	1	12.73	** < 0.001**
		Competition	1	17.15	** < 0.001**
		Origin × competition	1	0.06	0.800
Root biomass	log(y)	Origin	1	4.06	0.061
		Competition	1	325.85	** < 0.001**
		Origin × competition	1	11.83	** < 0.001**
SLA	log(y)	Origin	1	10.07	**0.007**
		Competition	1	26.97	** < 0.001**
		Origin × competition	1	2.49	0.118
Reproductive biomass	y	Origin	1	0.83	0.377
		Competition	1	188.08	** < 0.001**
		Origin × competition	1	0.00	0.965
Reproductive investment	log(y)	Origin	1	0.03	0.863
		Competition	1	24.88	** < 0.001**
		Origin × competition	1	0.03	0.862
Flower stem height	y	Origin	1	10.91	**0.004**
		Competition	1	5.54	**0.021**
		Origin × competition	1	0.01	0.934
Onset of flowering	y	Origin	1	0.97	0.339
		Competition	1	3.79	0.054
		Origin × competition	1	1.05	0.309

We used linear mixed effects models followed by ANOVA (Type 1). Response variables were transformed if needed to fulfill model assumptions. Shown are degrees of freedom (*df*), *F* values and *P* values, with significant *P* values (< 0.05) in bold

**Table 2:** Results of the statistical models testing the effects of temporal origin (ancestors, descendants), nutrient treatment (control, N, P, NP) and their interaction on the response variables (y) vegetative biomass, rosette diameter, root biomass, specific leaf area (SLA), reproductive biomass, reproductive investment, flower stem height and onset of flowering of *Leontodon hispidus*

Response variable	Transformation	Explanatory variable	*df*	*F* value	*P* value
Vegetative biomass	log(y)	Origin	1	3.54	0.081
		Nutrients	3	0.73	0.533
		Origin × nutrients	3	1.31	0.274
Rosette diameter	y	Origin	1	6.58	**0.024**
		Nutrients	3	6.18	** < 0.001**
		Origin × nutrients	3	0.44	0.722
Root biomass	sqrt(y)	Origin	1	11.53	**0.004**
		Nutrients	3	1.91	0.129
		Origin × nutrients	3	3.03	**0.031**
SLA	log(y)	Origin	1	5.25	**0.038**
		Nutrients	3	0.96	0.415
		Origin × nutrients	3	0.84	0.476
Reproductive biomass	log(y)	Origin	1	0.83	0.374
		Nutrients	3	2.31	0.079
		Origin × Nutrients	3	1.21	0.307
Reproductive investment	log(y)	Origin	1	0.00	0.965
		Nutrients	3	1.24	0.299
		Origin × nutrients	3	0.82	0.484
Flower stem height	y	Origin	1	8.83	**0.009**
		Nutrients	3	1.80	0.149
		Origin × nutrients	3	1.01	0.392
Onset of flowering	y	Origin	1	1.66	0.219
		Nutrients	3	3.55	**0.016**
		Origin × nutrients	3	0.06	0.982

We used linear mixed effects models followed by ANOVA (Type 1). Response variables were transformed if needed to fulfill model assumptions. Shown are degrees of freedom (*df*), *F* values and *P* values, with significant *P* values (< 0.05) in bold

## Results

**Fig. 2:**
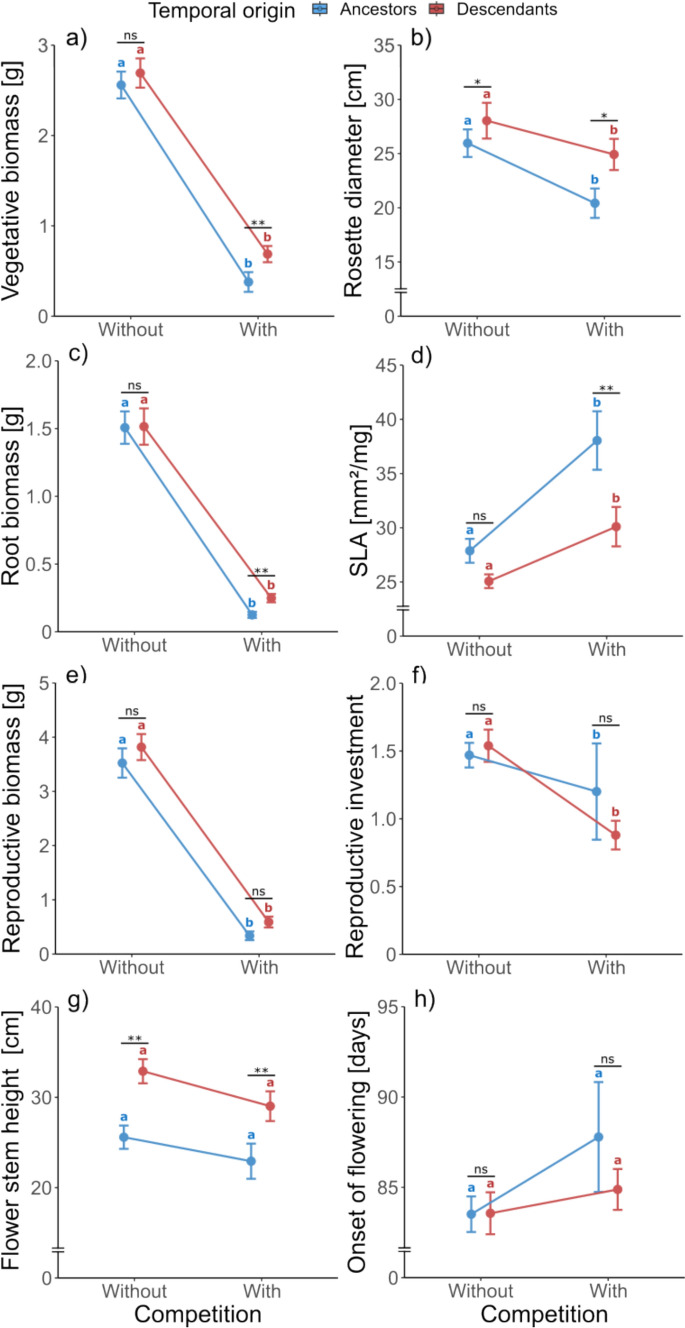
Vegetative biomass (**a**), rosette diameter (**b**), root biomass (**c**), specific leaf area (**d**), reproductive biomass (**e**), reproductive investment (**f**), flower stem height (**g**) and onset of flowering (**h**) of ancestors (blue) and descendants (red) of *Leontodon hispidus* grown either without competition or with competition (both without fertilizer). Shown are reaction norms connecting the means of the competition treatments with their standard errors. Significant differences between ancestors and descendants in each treatment are indicated with asterisks (*P* > 0.05 ns; *P* = 0.05–0.01*; *P* = 0.01–0.001**). Significant differences (*P* < 0.05) between competition treatments are shown by different letters in their respective color for each temporal origin (blue letters for ancestors and red letters for descendants)

According to the LMMs, the competition treatment had a significant effect on all measured traits except onset of flowering (Table 1). Competition generally decreased rosette diameter, reproductive biomass, and reproductive investment (Fig. 2b, e, f), but onset of flowering was not significantly affected by competition (Fig. 2h) and competition increased SLA (Fig. 2d). Significant differences between ancestors and descendants were found in vegetative biomass, rosette diameter, SLA (Specific Leaf Area), and flower stem height (Table 1). Irrespective of the competition treatment, the descendants had larger rosettes and taller flowering stems compared to ancestors (Fig. 2b, g). The effects of competition on vegetative biomass and root biomass differed between ancestors and descendants, as indicated by the significant interaction between competition and temporal origin (Table 1). Post-hoc comparisons showed that without competition, descendants and ancestors did not differ in their vegetative biomass or root biomass, but competition led to lower vegetative and root biomass in ancestors compared to descendants (Fig. 2a, c). Furthermore, descendants had a significantly lower SLA compared to ancestors under competition (Fig. 2d). Without competition, descendants had a significantly larger rosette diameter compared to ancestors (Fig. 2b) and taller flower stems (Fig. 2g) but did not differ in the remaining traits (Fig. 2).

**Fig. 3:**
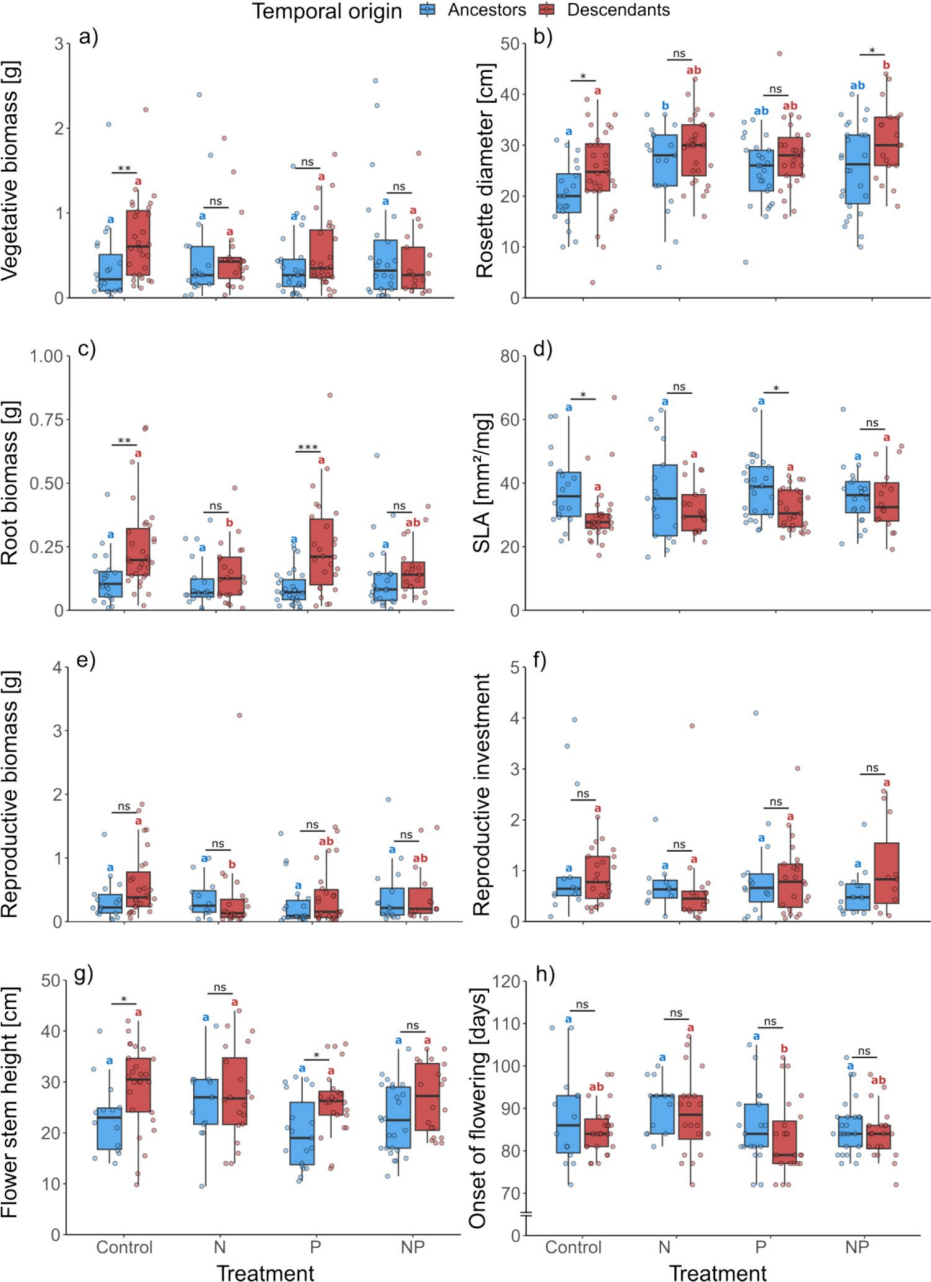
Vegetative biomass (**a**), rosette diameter (**b**), root biomass (**c**), specific leaf area (**d**), reproductive biomass (**e**), reproductive investment (**f**), flower stem height (**g**) and onset of flowering (**h**) of ancestors (blue) and descendants (red) of *Leontodon hispidus* grown under different nutrient treatments (control, N, P, NP; all with competition). Shown are boxplots with the raw data as scatter points. Significant differences between ancestors and descendants in each treatment are indicated with asterisks (*P* > 0.05 ns; *P* = 0.05–0.01*; *P* = 0.01–0.001**; *P* < 0.001***). Significant differences (*P* < 0.05) between nutrient treatments are shown by different letters in their respective color for each temporal origin separately (blue letters for ancestors and red letters for descendants)

The nutrient treatments significantly increased the rosette diameter (Table 2) and accelerated the onset of flowering (Table 2, Fig. 3). Descendants had significantly larger rosettes, larger root biomass, taller flower stems, and lower SLA compared to ancestors (Table 2). Reproductive biomass was also marginally significantly larger in descendants and we observed a marginally significant effect on vegetative biomass (Table 2). Post-hoc tests allowed to identify significant effects of specific nutrient treatments. Ancestors had larger rosettes in the N treatment compared to control, but not in the other two treatments. For descendants we detected significantly larger rosette diameter in the NP treatment only. (Fig. 3b). The N treatment decreased the root biomass and the reproductive biomass of descendants compared to the control (Fig. 3ce). No significant difference in root biomass could be detected between descendants and ancestors (Fig. 3c). Furthermore, descendants flowered later in the N treatment compared to the P treatment (Fig. 3h). According to the post-hoc comparisons, all significant differences between ancestors and descendants in the control treatment were canceled out by the N treatment. In the P treatment, descendants maintained higher root biomass (Fig. 3c), lower SLA (Fig. 3d) and taller flower stems (Fig. 3g) compared to ancestors, but no difference between temporal origins could be found in vegetative biomass and rosette diameter. In the NP treatment, descendants maintained their larger rosette diameter compared to ancestors (Fig. 3b). Finally, the differences of marginal *R*^2^ (*R*^2^_m_) and conditional *R*^2^ (*R*^2^_c_) were the highest in the flower stem height, but for all traits generally low in the competition dataset and more pronounced in the nutrient dataset (Online Resource 3).

## Discussion

To study the evolution of competitive ability and of responses to changing nutrient availability over the last decades, we conducted a resurrection study using ancestors collected in 1995 and descendants collected in 2018 after growing two refresher generations. We found evidence for evolution of increased competitive ability in descendants, as they showed better growth than ancestors when grown under competition. Furthermore, combining competition with nutrient treatments revealed that competitive ability also depended on the nutrient conditions.

### Evolution of competitive ability

Previous studies indicate that the competitive ability of plant populations and their evolution may be strongly affected by the highly diverse environmental changes over the last 25 years that include changes in climate (e.g., heatwaves and droughts), changes in nutrient availability, pollinator decline, and changes in grazing regime (Simon and Schmidt 2017, and references therein). In the nature reserve where our target plant had grown, the increasing dominance of the grass *B. pinnatum* (Bobbink and Willems 1987) or other competitors might have imposed strong selection pressure on rosette growing plants such as *L. hispidus* and, as a result, might have selected for an increased shade-avoidance response. The competition treatment in our experiment had a very strong effect on growth-related traits of *L. hispidus* (e.g., vegetative and root biomass). We observed that the competitor *B. pinnatum* was growing much taller than the rosettes of *L. hispidus*, which were substantially shaded as a consequence. Hence, *L. hispidus* received less light and competed for nutrients and space. Even though both ancestors and descendants were strongly affected by the competition, descendants outperformed ancestors for most growth-related traits (higher vegetative and root biomass, larger rosette diameter, taller flower stems) and maintained lower SLA. Notably, the larger rosette diameter of descendants did not trade off with leaf thickness, as indicated by the lower SLA. A low SLA may indicate a conservative strategy of *L. hispidus* by conserving resources under shading with large dry matter content, high concentration of cell walls, and high leaf longevity (Bennett et al. 2016).

Our results indicate that this population of *L. hispidus* has evolved traits to avoid shading by competitors and capture more light by increasing rosette diameter and flower stem height in the last 23 years. Such evolution has also been experimentally shown in *Impatiens capensis,* where directional selection on height and growth rate was caused by a competition treatment (McGoey and Stinchcombe 2009). Likewise, Weinig (2000) also reports stem elongation as an adaptation to competitive conditions in velvetleaf (*Abutilon theophrasti*). In the case of *L. hispidus,* the flower stems do not carry leaves, and therefore, their main function is to increase the attractiveness of the flowers for pollination and not capture light. As a consequence, avoiding shade may be crucial for this species to make the flowers more visible for pollinators, on which it is highly dependent as a self-incompatible species (Kühn and Klotz 2002). Additionally, the pollinator decline during the recent decades might affect the selection pressure of plants aboveground as plants compete for pollinators (Potts et al. 2010). The evolution of taller flower stems can be beneficial to better compete for pollinators by making the flowers more visible (Engel and Irwin 2003). Even though we did not study pollinator decline as a direct agent of selection, the evolution of taller flower stems would make sense in the context of pollinator decline during the recent decades (Potts et al. 2010). Competition for pollinators can also result in evolution of selfing (Eckert et al. 2010; Thomann et al. 2013), but the breakdown of self-incompatibility is often a slow process (Cheptou and Avendaño 2006; Lafuma and Maurice 2007) likely impeding a rapid increase of selfing in *L. hispidus*. In line with our findings, another resurrection study by Thomann and colleagues (2015) found evolution of larger flowers and flower longevity after 18 years in a population of the annual *Centaurea cyanus,* also a strongly self-incompatible species. Accordingly, it is possible that *L. hispidus* evolved other floral traits, such as capitula size, floral display, flower longevity, or flowering duration, which should be considered in future studies. However, our results cannot give definite proof that the increase in flowering stems is a specific adaptive change as it may be a response to other environmental stimuli or caused by non-adaptive evolution via gene drift, mutation, or recombination (Walsh and Lynch 2018). Furthermore, flowering stems might not have been the direct target of selection and might have been affected through pleiotropy of other traits such as leaf size. These could be addressed in future studies by combining the resurrection approach with genetic analyses, such as *Q*_ST_-*F*_ST_-analysis, to pinpoint if a trait was truly under selection (e.g., Rauschkolb et al. 2022). Another promising way to study the adaptive nature of observed changes is to transplant ancestors and descendants back to their original habitats where they have been collected to test whether descendant phenotypes show better performance under current environmental conditions than ancestor phenotypes, which would suggest adaptation (Karitter et al. 2024).

We expected that the evolution of higher belowground competitive ability would come at the expense of aboveground competitive ability due to a decrease in soil nutrient availability over the last decades. Indeed, we found compelling evidence that this population of *L. hispidus* has evolved higher competitive ability through faster growth belowground but also faster growth aboveground, making this population a stronger competitor for light and nutrients. Selection for increased competitive ability could either be facilitated directly by increased competition or indirectly by other selection agents that increase competitive ability as a side effect (e.g., low water availability selecting for faster root growth also makes plants more competitive belowground). It is possible that the environmental changes of the recent decades did not lead to a shift to more belowground competition, but that selection pressures equally increased below- and aboveground. Faster growth is especially important in the early life stage or early in the season, when interspecific shading is minimal. It was demonstrated that, fertilized environments with low disturbance are dominated by both, below- and above-ground competition (*i.e*., stress gradient hypothesis, Wilson and Tilman 1993). Furthermore, Wilson and Tilman (1993) showed that competition decreases as disturbance levels increase. The most prominent source of disturbance in our study population of *L. hispidus* is occasional sheep grazing. However, this level of disturbance might only be sufficient to reduce competitive pressure for a short period of time, and the plants will soon compete again with the fast growing grasses such as *B. pinnatum.* Finally, the competitor *B. pinnatum* may have also evolved to the changing environmental conditions, which could have led to a co-evolution with *L. hispidus* (Occhipinti 2013). An interesting approach to test for co-evolution would be to let ancestors and descendants of *L. hispidus* compete with ancestors and descendants of *B. pinnatum*. However, no ancestor accessions of *B. pinnatum* are available from our study site. In a similar vein, *B. pinnatum* may be an important but not the only competitor for *L. hispidus,* and the evolution of *L. hispidus* should be studied under more diverse competitive environments to support and complement our results.

### Responses to nutrient enrichment

The soil analyses of the original population site revealed a N content of 0.49%, which is comparable to other grasslands (Piqueray et al. 2011) and probably decreased in the studied site due to reduction of emissions since the 1990s (Klein et al. 2019; European Environment Agency 2021). The total P content, on the other hand, was 530 mg/kg in our studied site (Online Resource 2) and is much lower in comparison to other calcareous grasslands, which can reach over 1000 mg/kg of total P content (Alt et al. 2011; Wilson and Wheeler 2016). A possible explanation for the lower P content in the original site of *L. hispidus* could be that the slope of the site is increasing the runoff of nutrients, and thus, P is being washed out from the soil quickly and cannot accumulate in high quantities (Li et al. 2006).

While descendants generally outperformed ancestors without nutrient addition, adding nutrients generally reduced the differences between ancestors and descendants, which was most evident in the N and NP treatment, but less in the P treatment. Adding N removed all significant differences between ancestors and descendants compared to the control. This suggests that descendants have evolved an increased ability to compete for N since supplementing N no longer gives them an advantage due to decreased belowground competition (Newman 1973; Wilson and Tilman 1993). Nitrogen depositions decreased over the last three decades, and descendants might thus have evolved adaptations to lower N availability. This is further evidenced by the response of the descendants in the treatments with low N availability (control, P treatment), where we observe higher belowground competitive ability (i.e., higher root biomass) of descendants compared to ancestors. Chronic addition of nutrients (especially N) has been shown to decrease N use efficiency (NUE) of plants and has strong links to plant evolutionary history (Egan et al. 2019; Liao et al. 2021). Hence, it is likely that ancestors of *L. hispidus* also had evolved a low NUE due to high N emissions, while the subsequent decrease in emissions likely favored plants with higher NUE. While it remains challenging to pinpoint the main underlying selective agent, our results indicate that traits related to competitive ability are differentially expressed in response to nutrient availability in descendants. It is thus plausible that competitive ability evolved at least partly in response to changes in soil nutrient competition over the past 23 years.

We found significant differences between ancestors and descendants in the P treatment in several traits, such as larger root biomass or taller flower stems in descendants. However, these results did not differ from the control and therefore provide no evidence for evolution of P uptake strategies in the studied population. This finding is in line with the assumption that the availability of P did not significantly change in the recent decades and, therefore, did not act as a potential selection agent. Unfortunately, we did not measure plant available phosphorus in the natural population of our study species, which made it challenging to compare the nutrient concentrations with other studies in the same area (e.g., Janssen et al. 1998) and hindered precise assessments of the nutrient status of the studied grassland. Furthermore, we only used one fixed concentration for each nutrient treatment, whereas using multiple concentrations in an experiment would give more insight into underlying processes since plant responses might vary greatly depending on concentrations. We also applied the nutrient treatments only to plants growing under competition due to space constraints meaning that we cannot disentangle the interaction of competition and nutrient availability. Conducting a resurrection study using a full factorial design with competition and nutrient treatments could give further insights into the relationships between competition and nutrients as well as their evolution. Furthermore, we only used nine seed families in our study, which might not fully represent the genetic diversity of the population and therefore introduce bias that may correlate with certain trait characteristics. Accordingly, we might miss genotypes of the population with other adaptations. Furthermore, the analysis of *R*^2^_m_ and *R*^2^_c_ revealed a stronger influence of the seed family when comparing plants in different nutrient treatments, suggesting that there is genetic variation in the measured traits among seed families that is more strongly expressed under certain nutrient conditions. Finally, we only studied a single population of *L. hispidus*, making it difficult to generalize the results to the species level.

## Conclusion

In this study, we found evidence for evolutionary changes in competitive ability and responses to changes in nutrient availability in a population of *Leontodon hispidus*. Furthermore, supplementing nutrients (especially N) reduced differences in competitive ability between ancestors and descendants, suggesting that nutrients are a limiting factor in interspecific competition. We also found evolution of taller flower stems, which could be linked to pollinator decline as a means to increase the competitive ability for pollinator visits. Overall, the results of our study demonstrate the complexity of underlying processes of contemporary evolution and shed light on the importance of understudied potential selection agents that can be investigated using resurrection studies. Especially studying the effects of decreasing N emissions on plant populations after strong eutrophication will provide valuable insights for evolutionary responses of plant populations in future.

## Supplementary Information

Below is the link to the electronic supplementary material.

Supplementary file1 (PDF 263 KB)

Supplementary file2 (PDF 386 KB)

Supplementary file3 (PDF 194 KB)

## Data Availability

The data that support the findings of this study are available from Dryad [10.5061/dryad.hdr7sqvtn].
