# Supplementary material for: Evolution of competitive ability and the response to nutrient availability: a resurrection study with the calcareous grassland herb, Leontodon hispidus
Source: Oecologia. 2025 Jan 4;207(1):17. doi: 10.1007/s00442-024-05657-1 (PMC11700050; doi:10.1007/s00442-024-05657-1)
Supplement: Supplementary file 1 — Supplementary file1 (PDF 263 KB) [file 442_2024_5657_MOESM1_ESM.pdf]

Evolution of competitive ability and the response to nutrient availability: a resurrection study with the calcareous grassland herb, *Leontodon hispidus*

Oecologia

Pascal Karitter[1*], Emma Corvers[1], Marie Karrenbauer[1], Martí March-Salas[1], Bojana Stojanova[2], Andreas Ensslin[3], Robert Rauschkolb[4,5], Sandrine Godefroid[6], J.F. Scheepens[1]

[1]Plant Evolutionary Ecology, Institute of Ecology, Evolution and Diversity, Faculty of Biological Sciences, Goethe University Frankfurt, Max-von-Laue-Str. 13, 60438 Frankfurt am Main, Germany

[2] Department of Biology and Ecology, Faculty of Science, University of Ostrava, Chittussiho 10, CZ-710 00 Slezská Ostrava, Czech Republic

[3]Conservatory and Botanic Garden of the City of Geneva, Chemin de l´Impératrice 16 1, 1296 Chambésy, Geneva, Switzerland

[4]Institute of Ecology and Evolution with Herbarium Haussknecht and Botanical Garden, Department of Plant Biodiversity, Friedrich Schiller University Jena, Germany

[5]German Centre for Integrative Biodiversity Research (iDiv) Halle-Jena-Leipzig, Leipzig, Germany

[6]Meise Botanic Garden, Nieuwelaan 38, 1860 Meise, Belgium

[*] Corresponding author: Pascal Karitter (p.karitter@gmail.com; +4915175074964)

Online Resource 1

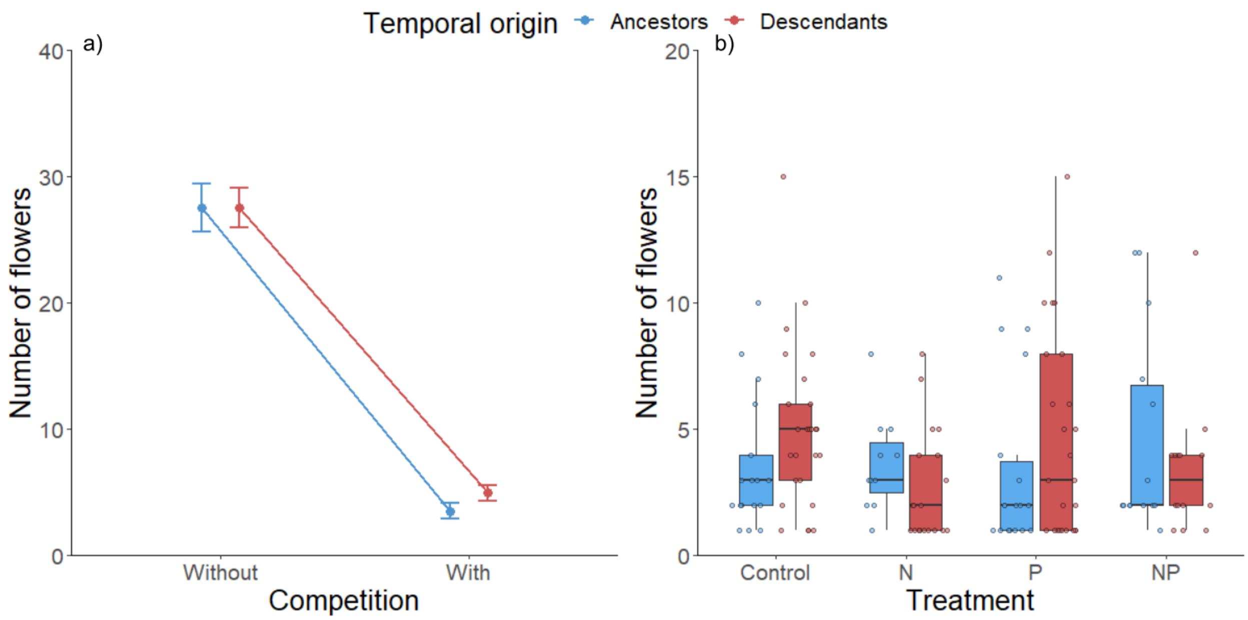

Online Resource 1 Number of flowers of ancestors (blue) and descendants (red) of *Leontodon hispidus* grown either without competition or with competition (a) and under different nutrient treatments (b). Shown are means and standard errors for (a) and boxplots with raw data as scatter points for (b)
