# Supplementary material for: Evolution of competitive ability and the response to nutrient availability: a resurrection study with the calcareous grassland herb, Leontodon hispidus
Source: Oecologia. 2025 Jan 4;207(1):17. doi: 10.1007/s00442-024-05657-1 (PMC11700050; doi:10.1007/s00442-024-05657-1)
Supplement: Supplementary file 2 — Supplementary file2 (PDF 386 KB) [file 442_2024_5657_MOESM2_ESM.pdf]

Evolution of competitive ability and the response to nutrient availability: a resurrection study with the calcareous grassland herb, *Leontodon hispidus*

Oecologia

Pascal Karitter[1*], Emma Corvers[1], Marie Karrenbauer[1], Martí March-Salas[1], Bojana Stojanova[2], Andreas Ensslin[3], Robert Rauschkolb[4,5], Sandrine Godefroid[6], J.F. Scheepens[1]

[1]Plant Evolutionary Ecology, Institute of Ecology, Evolution and Diversity, Faculty of Biological Sciences, Goethe University Frankfurt, Max-von-Laue-Str. 13, 60438 Frankfurt am Main, Germany

[2] Department of Biology and Ecology, Faculty of Science, University of Ostrava, Chittussiho 10, CZ-710 00 Slezská Ostrava, Czech Republic

[3]Conservatory and Botanic Garden of the City of Geneva, Chemin de l´Impératrice 16 1, 1296 Chambésy, Geneva, Switzerland

[4]Institute of Ecology and Evolution with Herbarium Haussknecht and Botanical Garden, Department of Plant Biodiversity, Friedrich Schiller University Jena, Germany

[5]German Centre for Integrative Biodiversity Research (iDiv) Halle-Jena-Leipzig, Leipzig, Germany

[6]Meise Botanic Garden, Nieuwelaan 38, 1860 Meise, Belgium

[*] Corresponding author: Pascal Karitter (p.karitter@gmail.com; +4915175074964)

Online Resource 2

Online Resource 2 Chemical composition of soil samples taken at the collection sites of our study species *Leontodon hispidus*. We took four samples of 25 cm² soil each at 10 cm depth at random positions and mixed them together. The samples were analyzed to determine the amount of fundamental minerals (total element content of P, K, S, Ca, total C, N and S), as well as pH level and salinity.

pH	Salinity [µS/cm]	N [%]	C [%]	TIC [%]	C org [%]	C/N	S [%]	Ca [mg/kg]	K [mg/kg]	Mg [mg/kg]	P [mg/kg]
6.46	88	0.49	6.90	0.00	6.90	14.14	0.01	3966	8608	1090	530
