# Supplementary material for: Evolution of competitive ability and the response to nutrient availability: a resurrection study with the calcareous grassland herb, Leontodon hispidus
Source: Oecologia. 2025 Jan 4;207(1):17. doi: 10.1007/s00442-024-05657-1 (PMC11700050; doi:10.1007/s00442-024-05657-1)
Supplement: Supplementary file 3 — Supplementary file3 (PDF 194 KB) [file 442_2024_5657_MOESM3_ESM.pdf]

Evolution of competitive ability and the response to nutrient availability: a resurrection study with the calcareous grassland herb, *Leontodon hispidus*

Oecologia

Pascal Karitter[1*], Emma Corvers[1], Marie Karrenbauer[1], Martí March-Salas[1], Bojana Stojanova[2], Andreas Ensslin[3], Robert Rauschkolb[4,5], Sandrine Godefroid[6], J.F. Scheepens[1]

[1]Plant Evolutionary Ecology, Institute of Ecology, Evolution and Diversity, Faculty of Biological Sciences, Goethe University Frankfurt, Max-von-Laue-Str. 13, 60438 Frankfurt am Main, Germany

[2] Department of Biology and Ecology, Faculty of Science, University of Ostrava, Chittussiho 10, CZ-710 00 Slezská Ostrava, Czech Republic

[3]Conservatory and Botanic Garden of the City of Geneva, Chemin de l´Impératrice 16 1, 1296 Chambésy, Geneva, Switzerland

[4]Institute of Ecology and Evolution with Herbarium Haussknecht and Botanical Garden, Department of Plant Biodiversity, Friedrich Schiller University Jena, Germany

[5]German Centre for Integrative Biodiversity Research (iDiv) Halle-Jena-Leipzig, Leipzig, Germany

[6]Meise Botanic Garden, Nieuwelaan 38, 1860 Meise, Belgium

[*] Corresponding author: Pascal Karitter (p.karitter@gmail.com; +4915175074964)

Online Resource 3

Online Resource 3 Conditional R^2 (R^2_c) and marginal R^2 (R^2_m) for the LMM models used in both the competition data subset and nutrient treatment subset

Response variable	Competition Dataset		Nutrient Dataset	
	R^2_c	R^2_m	R^2_c	R^2_m
Vegetative biomass	0.724	0.697	0.184	0.077
Rosette diameter	0.174	0.174	0.233	0.131
Root biomass	0.754	0.714	0.250	0.179
SLA	0.280	0.234	0.222	0.099
Reproductive biomass	0.657	0.623	0.105	0.084
Reproductive investment	0.208	0.208	0.069	0.051
Flower stem height	0.333	0.187	0.193	0.132
Onset of flowering	0.043	0.041	0.136	0.075
